# Supplementary material for: Comparative Analysis of Cholinergic Machinery in Carcinomas: Discovery of Membrane-Tethered ChAT as Evidence for Surface-Based ACh Synthesis in Neuroblastoma Cells
Source: Int J Mol Sci. 2025 Oct 23;26(21):10311. doi: 10.3390/ijms262110311 (PMC12608971; doi:10.3390/ijms262110311)
Supplement: Supplementary file 1 [file ijms-26-10311-s001.zip › Supplementary File 1_revised_Final.pdf]

International Journal of *Molecular Sciences*

Type of the Paper (Article, Review, Communication, etc.)

Comparative Analysis of Cholinergic Machinery in Carcinomas: *Discovery of Membrane-Tethered ChAT as Evidence for Surface-Based ACh Synthesis in Neuroblastoma Cells*

Banita Thakur*[1], Samar Tarazi*[1], Lada Doležalová[1], Homira Behbahani[1] and Taher Darreh-Shori[1]

[1]Department of Neurobiology, Care Sciences and Society; Division of Clinical Geriatrics, Center for Alzheimer Research. Karolinska Institutet, Sweden. Stockholm, Sweden.

* Correspondence: taher.darreh-shori@ki.se

Academic Editor: Firstname Lastname

Received: date
Revised: date
Accepted: date
Published: date

Citation: To be added by editorial staff during production.

Copyright: © 2025 by the authors. Submitted for possible open access publication under the terms and conditions of the Creative Commons Attribution (CC BY) license (https://creativecommons.org/licenses/by/4.0/).

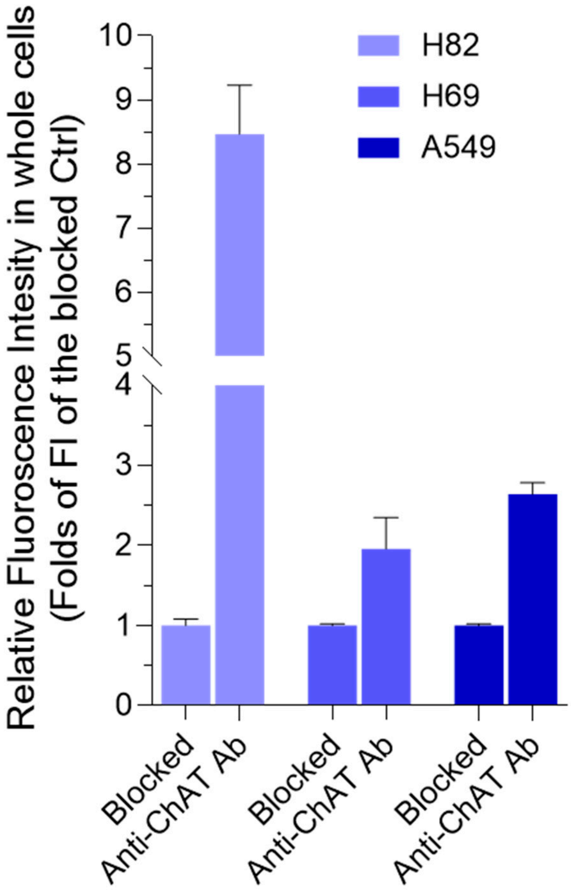

Figure S1. Relative fluorescence intensity (RFI) of anti-ChAT antibody compared to blocked antibody in H82, H69, and A549 cells. Whole-cell fluorescence intensity (FI) data from flow cytometry were used to calculate RFI, defined as the ratio of FI from cells stained with the anti-ChAT antibody to that from cells stained with the blocked antibody. Data are presented as fold differences (Mean ± SD). The FI of H82, H69, and A549 cells stained with the anti-ChAT antibody was approximately 8.5-, 2.0-, and 2.5-fold higher, respectively, than that of cells incubated with the blocked antibody.

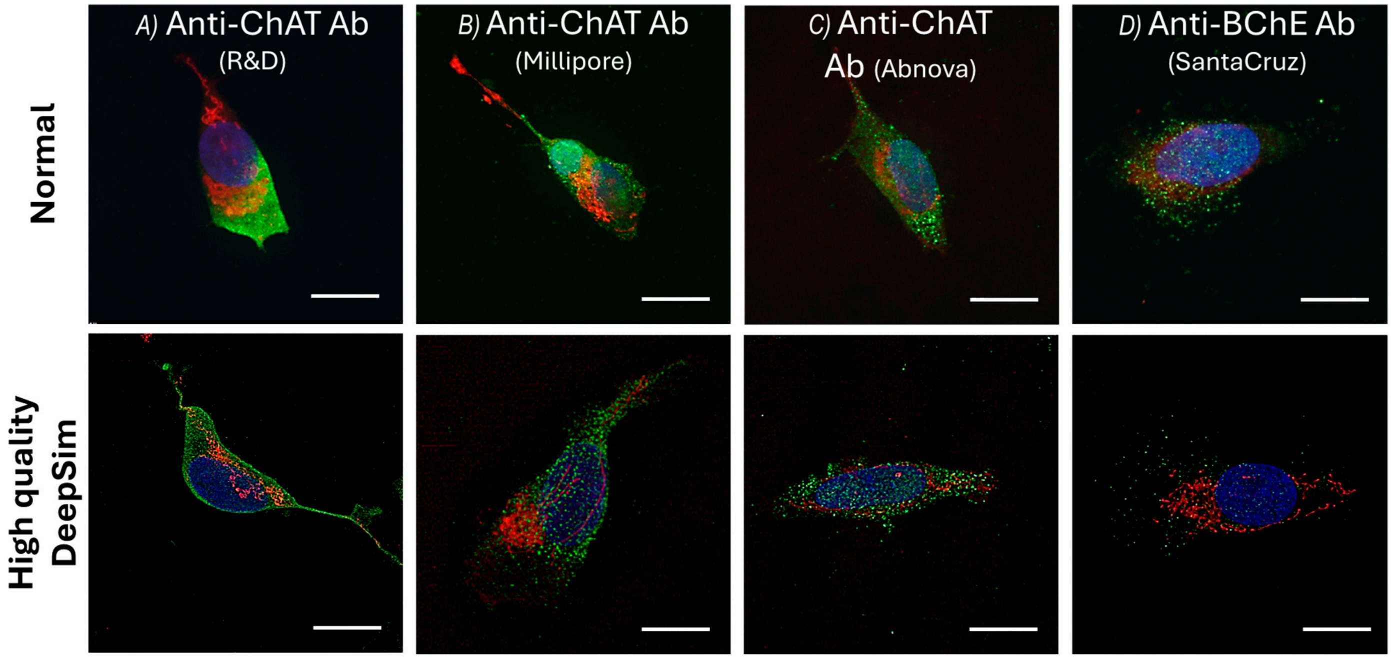

Figure S2. Surface (extracellular) immunostaining of ChAT and BChE in SH-SY5Y neuroblastoma cells using different antibodies. Staining was performed on ice following cell fixation, without permeabilization, to selectively label surface-exposed epitopes. Representative super-resolution immunofluorescence images show staining with three independent anti-ChAT antibodies (A–C, green) and one anti-BChE antibody (D, green), alongside intracellular co-labeling of mitochondria (Mitotracker Red, red) and nuclei (DAPI, blue). The upper panels display standard confocal images, while the lower panels show high-resolution images acquired using DeepSIM technology (CrestOptics) at 60× magnification. Scale bar: 10 µm. Primary antibodies used: mouse anti-ChAT (MAB3447, R&D Systems), rabbit polyclonal anti-ChAT (AB143, Millipore), rabbit polyclonal anti-ChAT (PAB14536, Abnova), and mouse monoclonal anti-BChE (D-5, sc-377403, Santa Cruz).

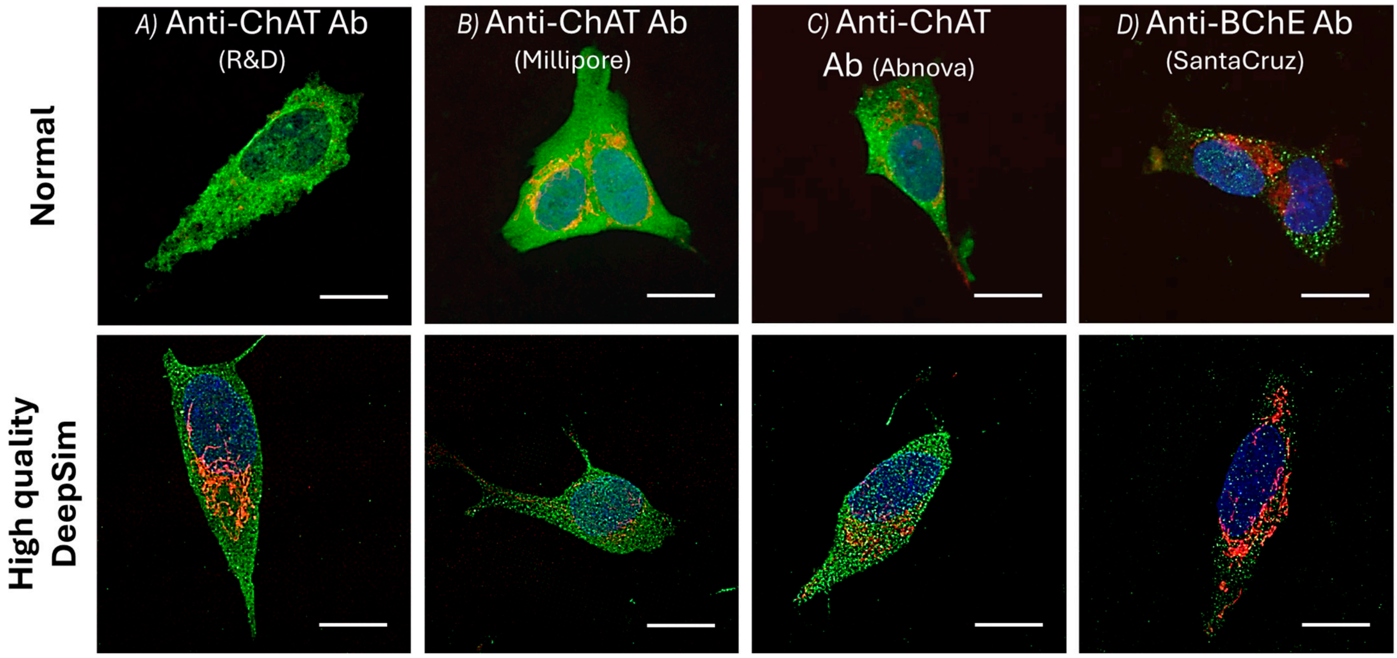

Figure S3. Extracellular and intracellular (whole-cell) immunostaining of ChAT and BChE in SH-SY5Y neuroblastoma cells. Cells were first fixed and stained on ice without permeabilization to label extracellular epitopes. Following a second fixation, cells were permeabilized to allow access to intracellular targets and re-incubated with the same antibodies. Representative super-resolution immunofluorescence images show staining with three different anti-ChAT antibodies (A–C, green) and one anti-BChE antibody (D, green), co-labeled with mitochondrial marker Mitotracker Red (red) and nuclear stain DAPI (blue). The upper panels show standard confocal images, and the lower panels present DeepSIM high-resolution images acquired at 60× magnification. Scale bar: 10 µm. Primary antibodies used: mouse anti-ChAT (MAB3447, R&D Systems), rabbit polyclonal anti-ChAT (AB143, Millipore), rabbit polyclonal anti-ChAT (PAB14536, Abnova), and mouse monoclonal anti-BChE (D-5, sc-377403, Santa Cruz).

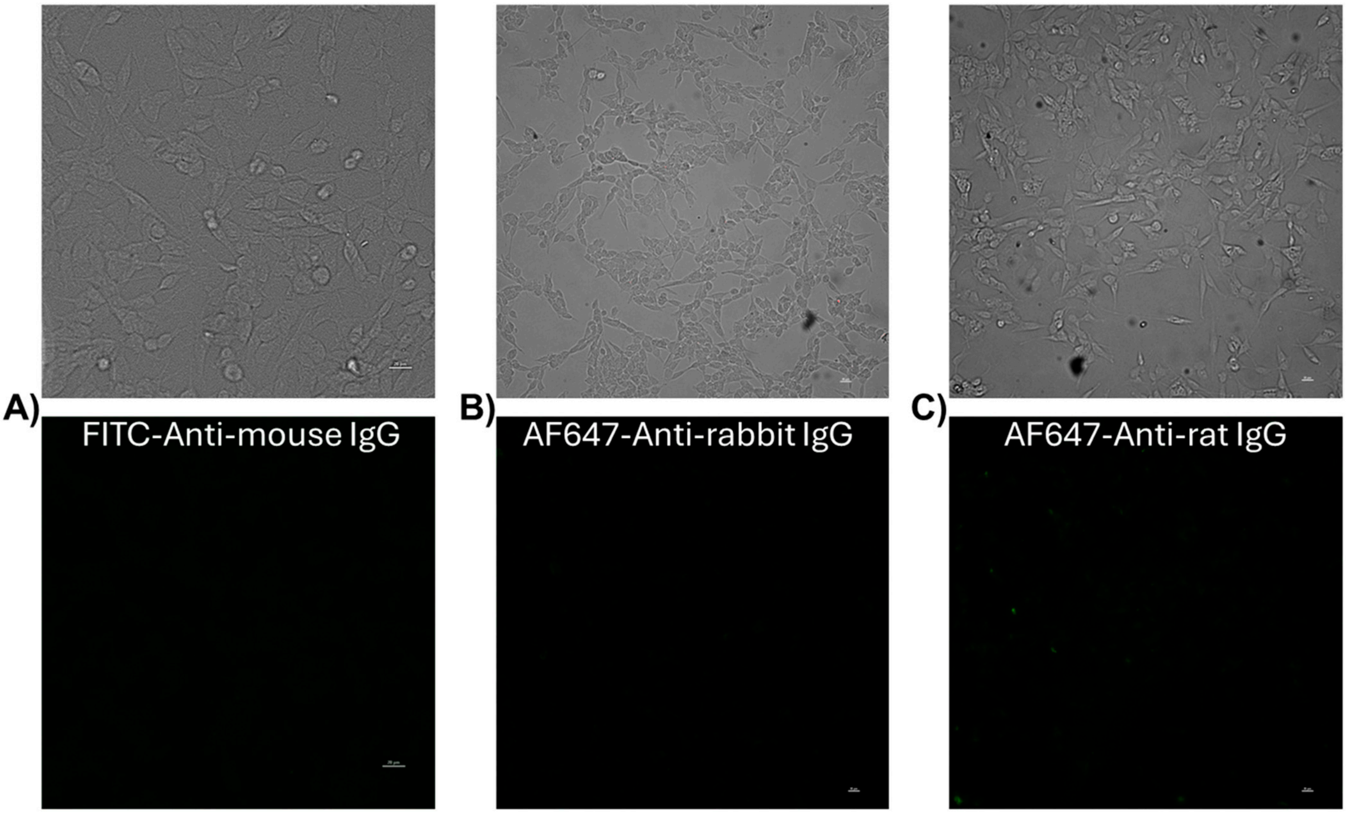

Figure S4. Negative controls for secondary antibody staining in SH-SY5Y neuroblastoma cells. Bright-field images of SH-SY5Y cells are followed by negative immunofluorescence controls of the same field of view, where cells were incubated with PBS instead of primary antibodies and exposed only to secondary antibodies. Panels show staining with: (A) FITC-conjugated goat anti-mouse IgG, (B) Alexa Fluor 647-conjugated goat anti-rabbit IgG, and (C) Alexa Fluor 647-conjugated goat anti-rat IgG. All secondary antibodies were obtained from Invitrogen. Magnification: 20×; Scale bar: 20 μm.
