# Supplementary material for: Comparative Analysis of Cholinergic Machinery in Carcinomas: Discovery of Membrane-Tethered ChAT as Evidence for Surface-Based ACh Synthesis in Neuroblastoma Cells
Source: Int J Mol Sci. 2025 Oct 23;26(21):10311. doi: 10.3390/ijms262110311 (PMC12608971; doi:10.3390/ijms262110311)
Supplement: Supplementary file 1 [file ijms-26-10311-s001.zip › Supplementary File 2_The 3D video images.pptx]

## Slide 1
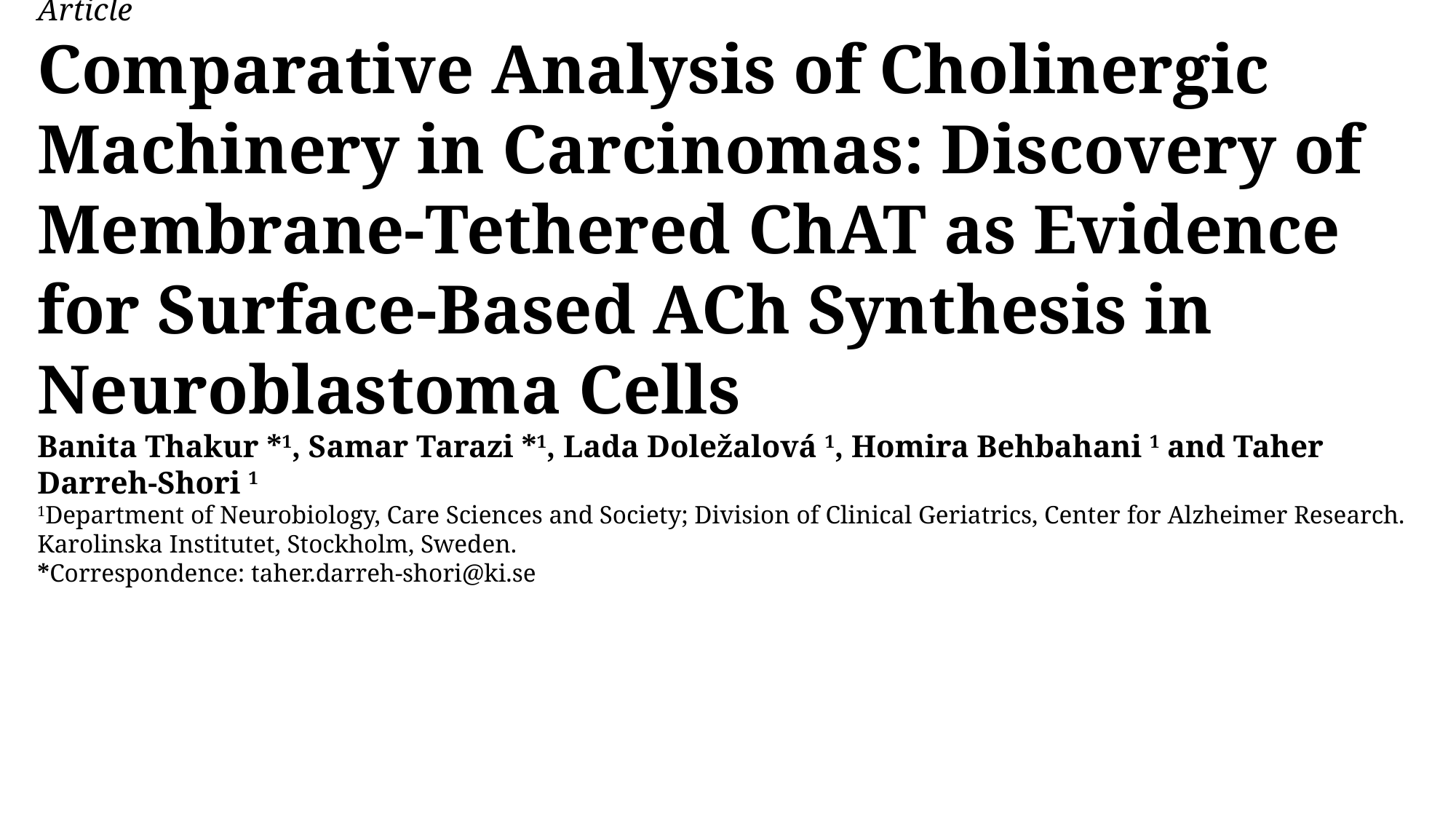

# Article
Comparative Analysis of Cholinergic Machinery in Carcinomas: Discovery of Membrane-Tethered ChAT as Evidence for Surface-Based ACh Synthesis in Neuroblastoma Cells
Banita Thakur *1, Samar Tarazi *1, Lada Doležalová 1, Homira Behbahani 1 and Taher Darreh-Shori 1
1Department of Neurobiology, Care Sciences and Society; Division of Clinical Geriatrics, Center for Alzheimer Research. Karolinska Institutet, Stockholm, Sweden.
*Correspondence: taher.darreh-shori@ki.se

## Slide 2
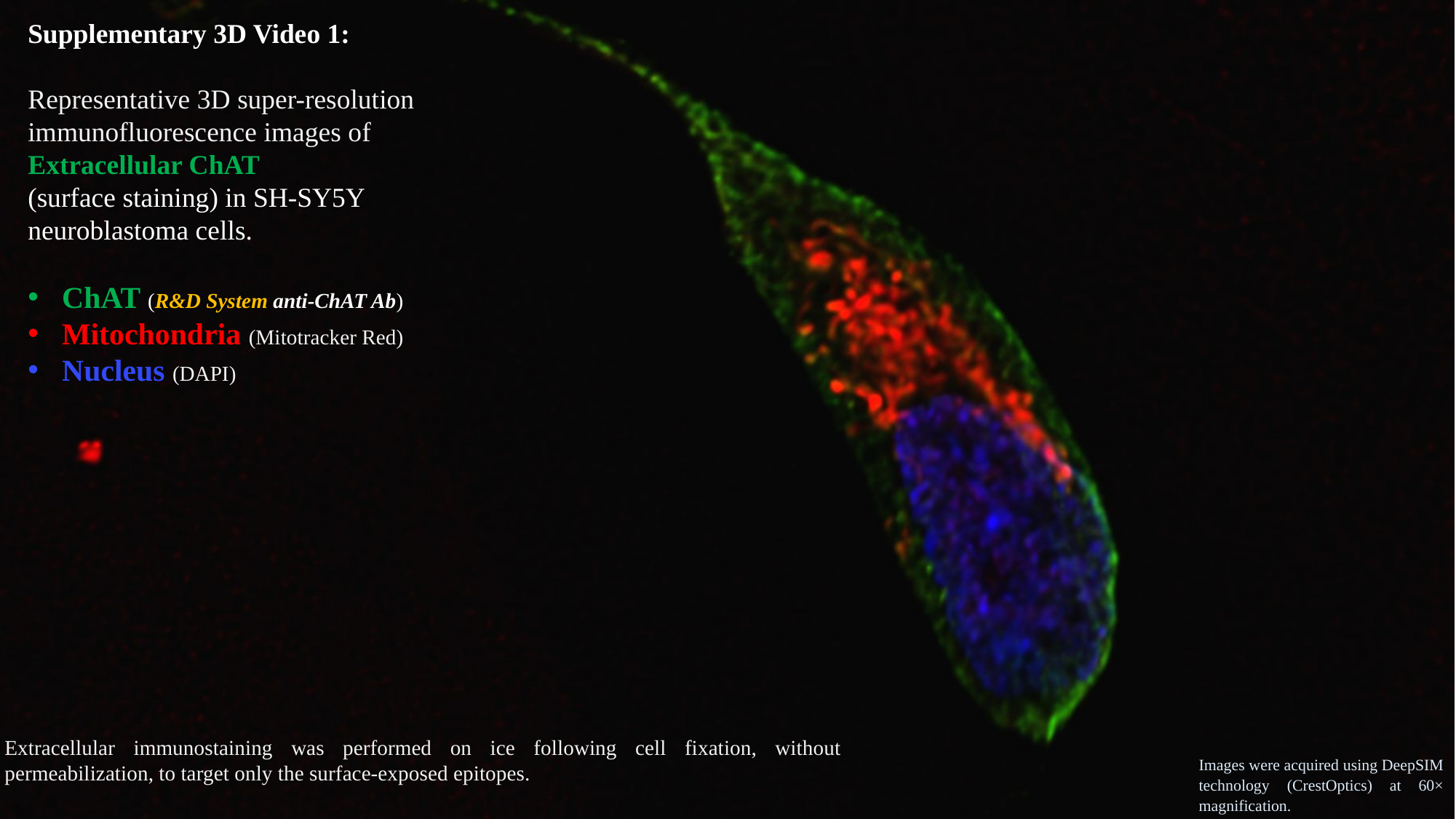

3D Videos and Images of Surface ChAT staining (R&D anti-ChAT Ab)
Supplementary 3D Video 1:
Representative 3D super-resolution immunofluorescence images of
Extracellular ChAT
(surface staining) in SH-SY5Y neuroblastoma cells.
ChAT (R&D System anti-ChAT Ab)
Mitochondria (Mitotracker Red)
Nucleus (DAPI)
Extracellular immunostaining was performed on ice following cell fixation, without permeabilization, to target only the surface-exposed epitopes.
Images were acquired using DeepSIM technology (CrestOptics) at 60× magnification.

## Slide 3
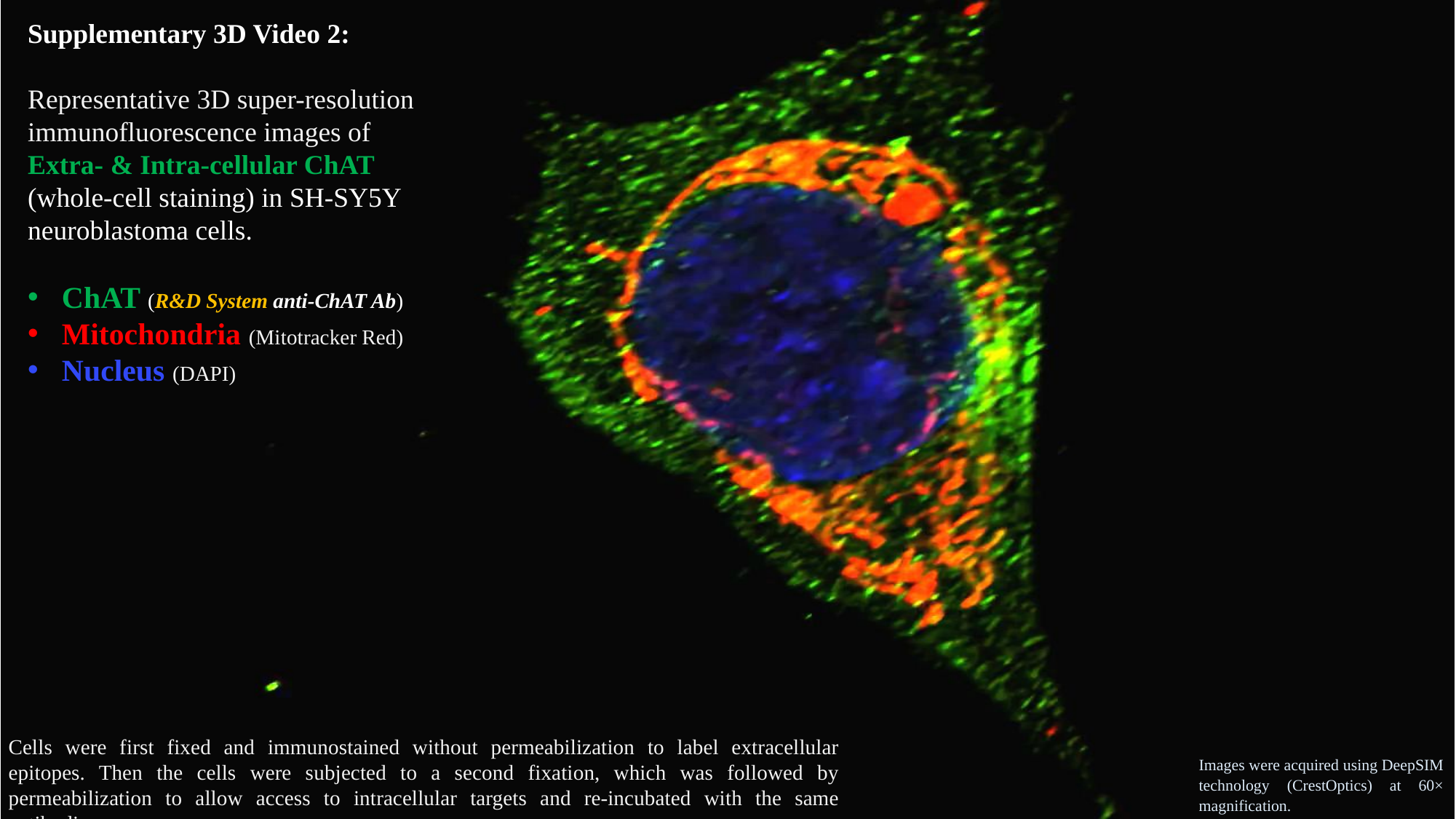

3D Videos and Images of Whole-cell ChAT staining (R&D anti-ChAT Ab)
Supplementary 3D Video 2:
Representative 3D super-resolution immunofluorescence images of
Extra- & Intra-cellular ChAT (whole-cell staining) in SH-SY5Y neuroblastoma cells.
ChAT (R&D System anti-ChAT Ab)
Mitochondria (Mitotracker Red)
Nucleus (DAPI)
Cells were first fixed and immunostained without permeabilization to label extracellular epitopes. Then the cells were subjected to a second fixation, which was followed by permeabilization to allow access to intracellular targets and re-incubated with the same antibodies.
Images were acquired using DeepSIM technology (CrestOptics) at 60× magnification.

## Slide 4
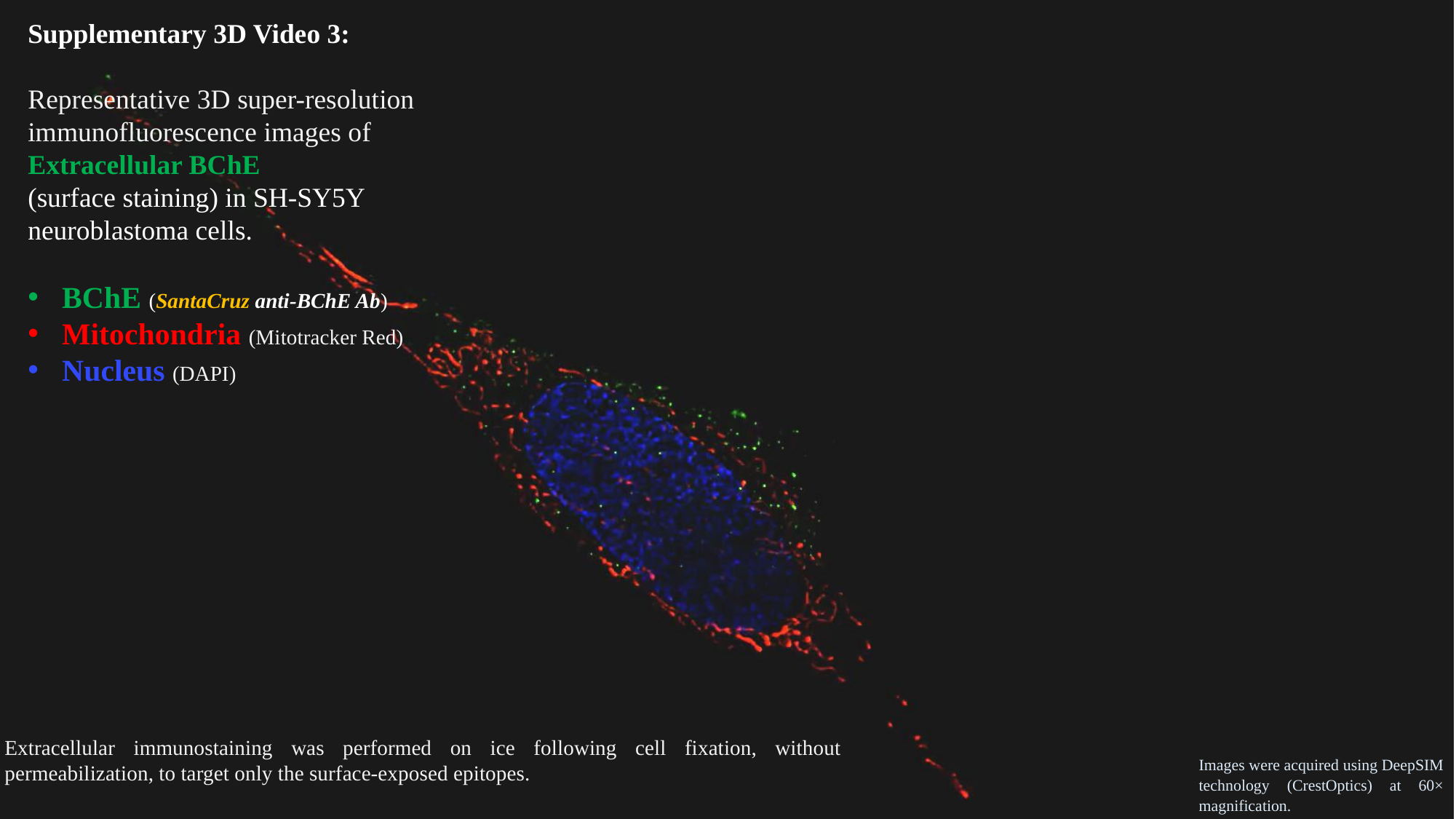

3D Videos and Images of Surface BChE staining
Supplementary 3D Video 3:
Representative 3D super-resolution immunofluorescence images of
Extracellular BChE
(surface staining) in SH-SY5Y neuroblastoma cells.
BChE (SantaCruz anti-BChE Ab)
Mitochondria (Mitotracker Red)
Nucleus (DAPI)
Extracellular immunostaining was performed on ice following cell fixation, without permeabilization, to target only the surface-exposed epitopes.
Images were acquired using DeepSIM technology (CrestOptics) at 60× magnification.

## Slide 5
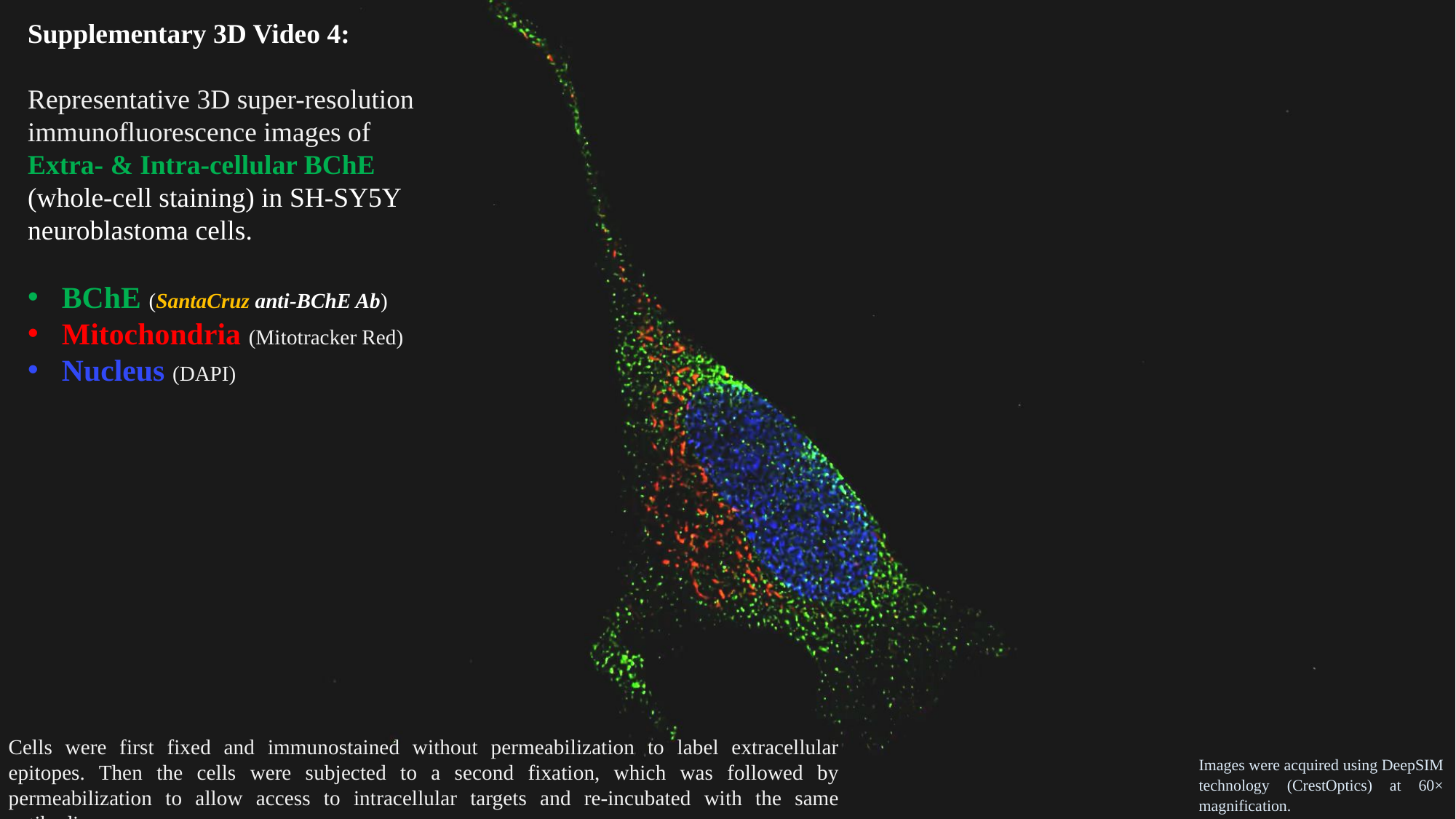

3D Videos and Images of Whole-cell BChE staining
Supplementary 3D Video 4:
Representative 3D super-resolution immunofluorescence images of
Extra- & Intra-cellular BChE (whole-cell staining) in SH-SY5Y neuroblastoma cells.
BChE (SantaCruz anti-BChE Ab)
Mitochondria (Mitotracker Red)
Nucleus (DAPI)
Cells were first fixed and immunostained without permeabilization to label extracellular epitopes. Then the cells were subjected to a second fixation, which was followed by permeabilization to allow access to intracellular targets and re-incubated with the same antibodies.
Images were acquired using DeepSIM technology (CrestOptics) at 60× magnification.
3D Videos and Images of Whole cell BChE staining

## Slide 6
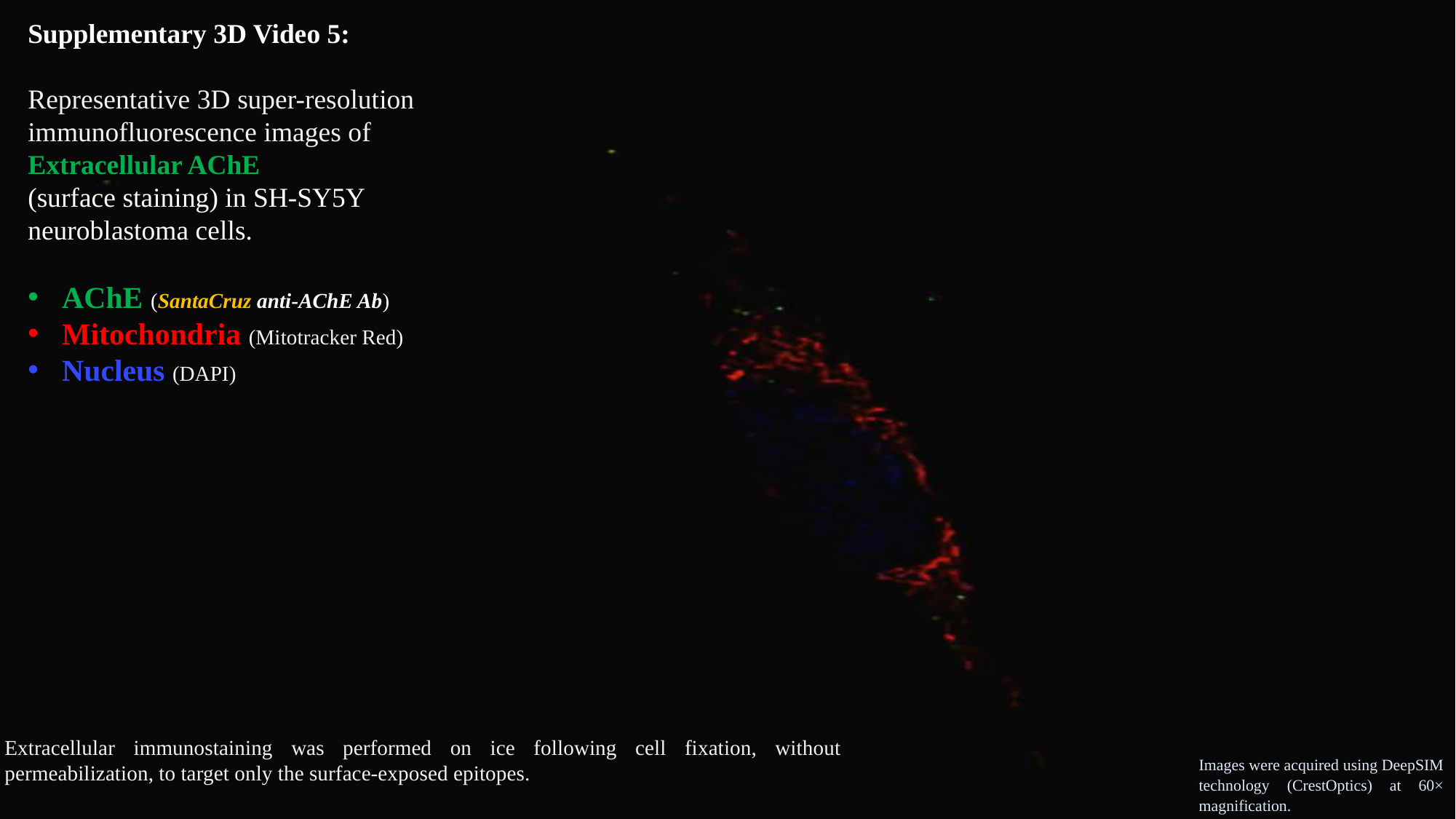

3D Videos and Images of Surface AChE staining
Supplementary 3D Video 5:
Representative 3D super-resolution immunofluorescence images of
Extracellular AChE
(surface staining) in SH-SY5Y neuroblastoma cells.
AChE (SantaCruz anti-AChE Ab)
Mitochondria (Mitotracker Red)
Nucleus (DAPI)
Extracellular immunostaining was performed on ice following cell fixation, without permeabilization, to target only the surface-exposed epitopes.
Images were acquired using DeepSIM technology (CrestOptics) at 60× magnification.
3D Videos and Images of Surface AChE staining

## Slide 7
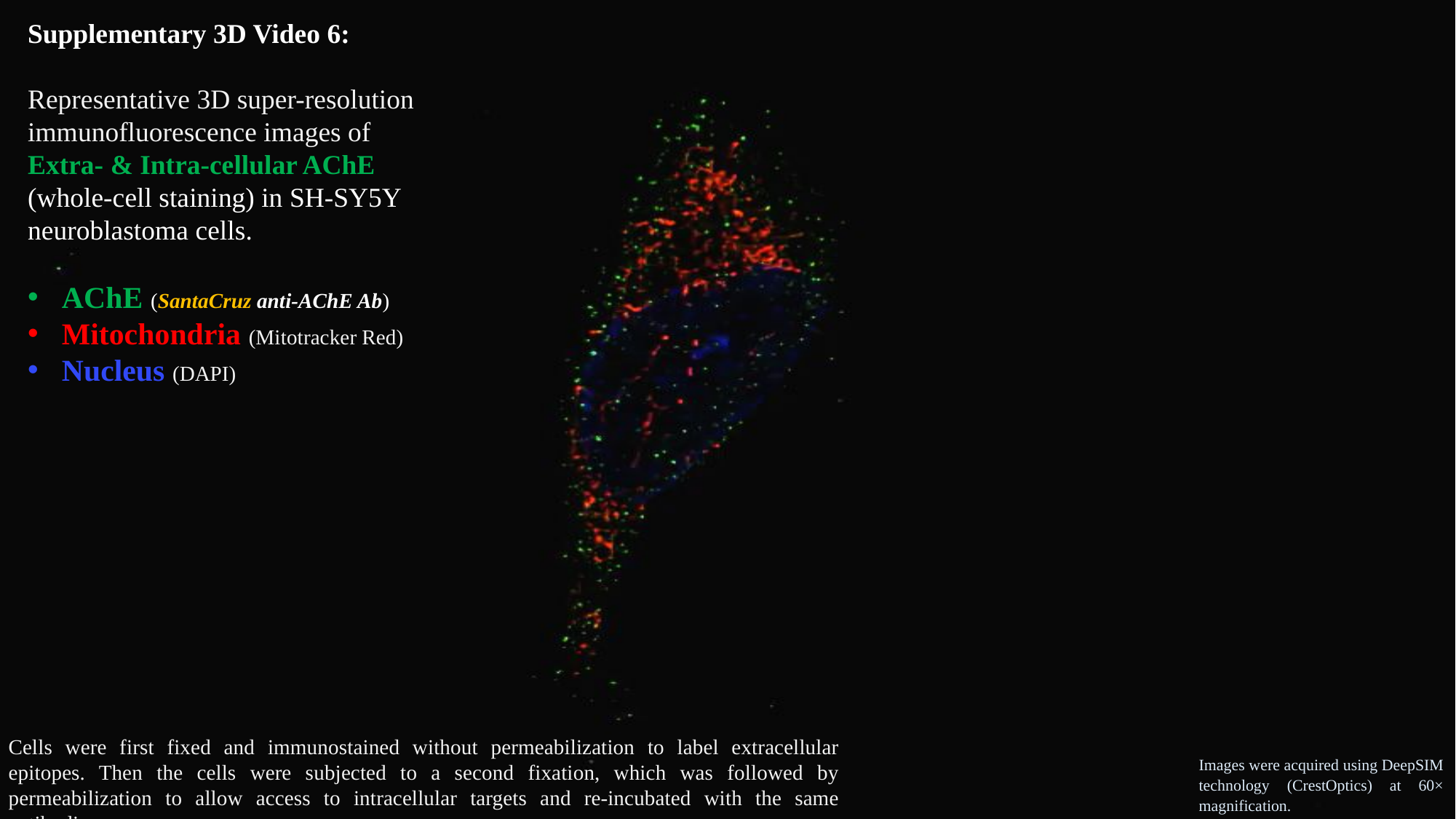

3D Videos and Images of Whole cell AChE staining
Supplementary 3D Video 6:
Representative 3D super-resolution immunofluorescence images of
Extra- & Intra-cellular AChE (whole-cell staining) in SH-SY5Y neuroblastoma cells.
AChE (SantaCruz anti-AChE Ab)
Mitochondria (Mitotracker Red)
Nucleus (DAPI)
Cells were first fixed and immunostained without permeabilization to label extracellular epitopes. Then the cells were subjected to a second fixation, which was followed by permeabilization to allow access to intracellular targets and re-incubated with the same antibodies.
Images were acquired using DeepSIM technology (CrestOptics) at 60× magnification.
